# Partially ordered state of ice XV

**DOI:** 10.1038/srep28920

**Published:** 2016-07-04

**Authors:** K. Komatsu, F. Noritake, S. Machida, A. Sano-Furukawa, T. Hattori, R. Yamane, H. Kagi

**Affiliations:** 1 Geochemical Research Center, Graduate School of Science, The University of Tokyo, Hongo 7-3-1, Bunkyo-ku, Tokyo 113-0033, Japan; 2 CROSS-Tokai, Research Center for Neutron Science and Technology, IQBRC Bldg, 162-1 Shirakata, Tokai, Ibaraki 319-1106, Japan; 3 J-PARC Center, Japan Atomic Energy Agency, 2-4 Shirakata-Shirane, Tokai, Ibaraki 319-1195, Japan

## Abstract

Most ice polymorphs have order–disorder “pairs” in terms of hydrogen positions, which contributes to the rich variety of ice polymorphs; in fact, three recently discovered polymorphs— ices XIII, XIV, and XV—are ordered counter forms to already identified disordered phases. Despite the considerable effort to understand order–disorder transition in ice crystals, there is an inconsistency among the various experiments and calculations for ice XV, the ordered counter form of ice VI, *i.e.*, neutron diffraction observations suggest antiferroelectrically ordered structures, which disagree with dielectric measurement and theoretical studies, implying ferroelectrically ordered structures. Here we investigate *in-situ* neutron diffraction measurements and density functional theory calculations to revisit the structure and stability of ice XV. We find that none of the completely ordered configurations are particular favored; instead, partially ordered states are established as a mixture of ordered domains in disordered ice VI. This scenario in which several kinds of ordered configuration coexist dispels the contradictions in previous studies. It means that the order–disorder pairs in ice polymorphs are not one-to-one correspondent pairs but rather have one-to-*n* correspondence, where there are *n* possible configurations at finite temperature.

Ice crystals show tremendous structural variety—with the recent discovery of ice XVI^1^, there are now 17 known crystalline polymorphs if ices Ih and Ic are distinguished from each other^2^,^3^. The variety of ice structures may be explained from two viewpoints: 1) Variety of O sublattices—a number of tetrahedral configurations of water molecules have similar enthalpy and therefore can thus stably exist over some pressure–temperature (*p*–*T*) ranges or metastably appear through a particular *p*–*T* path. 2) Variety of H sublattices—each O sublattice has pairs of hydrogen disorder–order configurations (i.e., ices Ih–XI, III–IX, V–XIII, VI–XV, VII–VIII, and XII– XIV) caused by subtle reduction of enthalpy and entropy for ordering or gain for disordering. Consequently, most ice phases except ice X are found in a narrow *p*–*T* region, which could hinder the understanding of the relative stability relations of respective ice phases.

Ice XV, the ordered form of ice VI, was identified by Salzmann *et al*.^4^. They observed neutron diffraction for ice XV prepared from ice VI by slow cooling from 250 to 80 K at 0.2 K/min and ~0.9 GPa and found several additional peaks, which should have been absent owing to the symmetry of ice VI. In their study, detailed structure and stability investigations were carried out on a sample recovered at ambient pressure, and they showed that the transition temperature between ice VI and XV is about 130 K at ambient pressure. They also noted that the transition temperature might not alter even at high pressure because the volume change (*ΔV*) accompanying the transition was sufficiently small such that the phase boundary could be flat in terms of pressure based on the Clausius–Clapeyron relation (*dT*/*dP* = *ΔV*/*ΔS*). Note that the ice VI-XV phase boundary under pressure was not experimentally confirmed in their study and could still be controversial because the entropy change (*ΔS*) would also be rather small; it should be less than the Pauling entropy^5^ (*R* ln (3/2) ≅ 3.37 J mol^−1^ K^−1^) as the hydrogen ordering is incomplete and only occurs partially^4^.

Prior to the identification of ice XV by Salzmann *et al*., the phenomena suggesting hydrogen ordering in ice VI had been reported in a number of studies using neutron diffraction^6^, and dielectric^7^, thermal expansion^8^, heat capacity^9^, and Raman spectroscopy^10^ measurements. Several theoretical calculations regarding hydrogen ordering in ice VI^11^,^12^ have also been reported. As Salzmann *et al*. noted in their review paper^13^, the results from two neutron diffraction studies^4^,^6^ showing antiferroelectrically ordered states conflict with those from dielectric measurement^7^ and theoretical calculations^11^,^12^, suggesting ferroelectric ordering. Their review paper triggered more experimental^14^,^15^ and theoretical^16^,^17^ studies. For example, density functional theory (DFT) calculations^16^ and Raman spectra assignment by DFT calculations^15^ suggested an antiferroelectrically ordered structure with space group 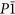, supporting the neutron diffraction results^4^. Conversely, the most recent calculations^17^ reconfirmed that the ferroelectrically ordered structure with space group *Pc*^18^ has the least energy, and the energy would be dependent on the surrounding matrix as discussed later. Despite considerable effort, this inconsistency has not been resolved and the reason for the conflict has yet to be fully explained. Thus, the following points are at issue: (i) few low-*T*, high-*p in-situ* neutron results have been reported, probably because of technical difficulties or insufficient data quality; (ii) all possible ordered configurations satisfying the Bernal–Fowler ice rules (simply called the “ice rules” hereafter) were not fully considered or sometimes confused (see arguments in Del Ben *et al*.)^17^ in previous studies; and (iii) it is difficult to analyze partially ordered states via theoretical studies. The technical difficulties in measuring neutron diffraction under non-ambient conditions have been overcome by recent significant progress of neutron facilities^19^ using originally developed equipment for sample environments^20^. Thus, it is now appropriate to reinvestigate the structure and stability of ice XV and to present a consistent explanation for the conflicts among previous studies.

## Results

### Stability of ice XV

**Figure 1:**
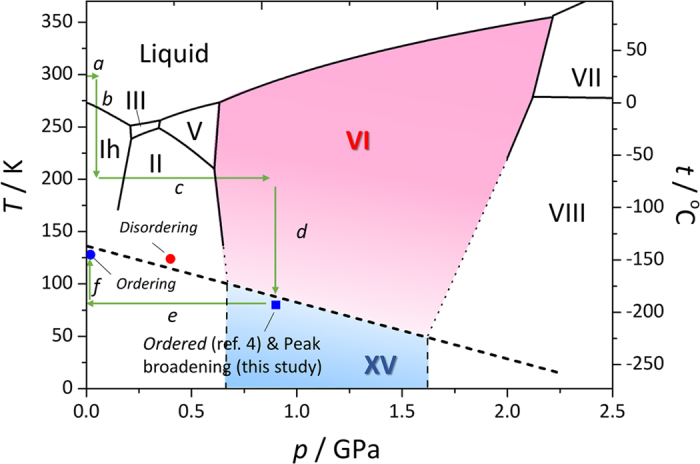
Proposed phase boundary between ice VI and XV. Other phase boundaries are based on previous studies compiled by Dunaeva *et al*.^51^. A typical pressure–temperature path in this study is shown with green arrows as follows: (**a**) Apply a load just enough to seal the sample (*i.e.*, a few tons), (**b**) cool down to 200 K at ~10 K/min, (**c**) compress up to 1.0–1.7 GPa to make fine powder of ice VI through several phase transitions, (**d**) cool down to ~80 K at 0.2 K/min, (**e**) download to ambient pressure, and (**f**) heat up to ~128 K. The phase boundary of ices IV and XV has a slightly negative slope, which is constrained by results from our neutron diffraction experiments denoted as blue (ordering) and red (disordering) circles. The observed peak broadening and the result from a previous study^4^ are denoted with a blue square.

**Figure 2:**
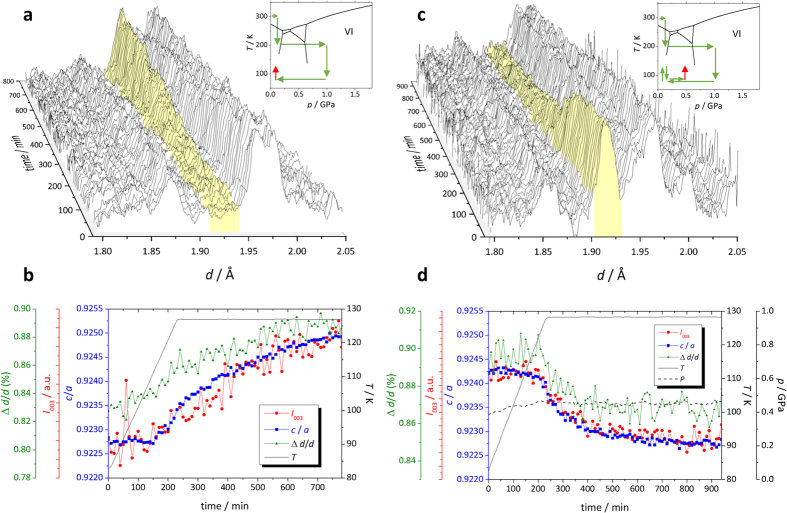
Neutron diffraction patterns and refined parameters with *p*–T conditions. (**a**) Selected area of neutron diffraction patterns showing the growth of the 003 reflection (shaded area) as a function of time, indicating the hydrogen ordering process from ice VI to XV for the sample recovered from 1.0 to 0 GPa with increasing and then stable temperature. The detailed *p*–*T* path is shown in the inset as arrows, with red arrows illustrating the paths for collected data. (**b**) Refined parameters corresponding to (**a**), the intensities for 003 reflection (*I*_003_), the axial ratio of lattice parameters (*c*/*a*), and the peak width divided by *d*-value (Δ*d*/*d*). The averaged temperature measured by two K-type thermocouples attached on the anvils is also shown. (**c**) Neutron diffraction patterns showing back transition to disordered ice VI. The *p*–*T* path is continued from (**a**), and compression up to 0.4 GPa and increasing temperature, as shown as the inset arrows. (**d**) Refined parameters corresponding to (**c**), with pressure and temperature variations.

The phase transition from ice VI to XV can be detected by neutron diffraction as the appearance of additional peaks that are absent in ice VI and also as an elongation of the *c*-axis, as reported by Salzmann *et al*.^4^. Accordingly, we expected that these phenomena would be observed under pressure as well as under ambient pressure. However, when the sample temperature was decreased from 200 to 80 K (path *d*, Fig. 1) at a rate of 0.2 K/min at high pressures (0.9–1.6 GPa) over 5 runs, neither additional peaks nor lattice elongation was observed (Supplementary Fig. S1). Rietveld refinements also showed that the disordered ice VI model does fit well to the obtained neutron diffraction patterns (see Supplementary Fig. S2). We also measured neutron diffraction after decreasing pressure to ~0.4 GPa at temperatures varying from 80 to 130 K, but again, signs of ordering were not observed (Supplementary Fig. S3). This disparity with previous studies may be caused by the kinetic effects owing to insufficient doping, as discussed in detail later. Instead, after recovery to ambient pressure and heating up to 128 K (path *f*), additional peaks and lattice elongation were observed (Fig. 2a). We realized that the *a*-axis is not significantly affected by ordering but instead simply expands or shrinks with varying temperature, whereas the *c*-axis is affected by both hydrogen ordering and thermal expansion. In this case, the *c*/*a* ratio would be a good order parameter because the effect of thermal expansion on the *c*-axis change is cancelled out by that on the *a*-axis. The intensities of additional peaks (e.g., 003) correspond closely to changes in *c*/*a* (Fig. 2b). Note that the appearance of additional peaks and change in *c*/*a* were observed at ~110 K in this case and that additional peaks were still being grown after nearly 10 h at a constant temperature of 128 K.

This result shows that the ordering process is so sluggish that it is not observable below 110 K and never completes over the experimental time scale as found in the previous study^14^. The obtained partially ordered ice XV was quenched to 84 K, compressed again to 0.4 GPa, and heated up to 127 K, where it was maintained. At this point, the opposite phenomena were observed: the intensities of additional peaks and magnitude of *c*/*a* started to decrease at 124 K and kept decreasing at 127 K. Again, the peak intensity and *c/a* were closely correlated (Fig. 2c,d). The pressure was slightly increased from 0.40 to 0.45 GPa with increasing temperature during this process. This clearly shows that disordered ice VI is more stable than ice XV at 0.45 GPa and 124 K, thereby showing a negative phase boundary between ices VI and XV.

**Figure 3:**
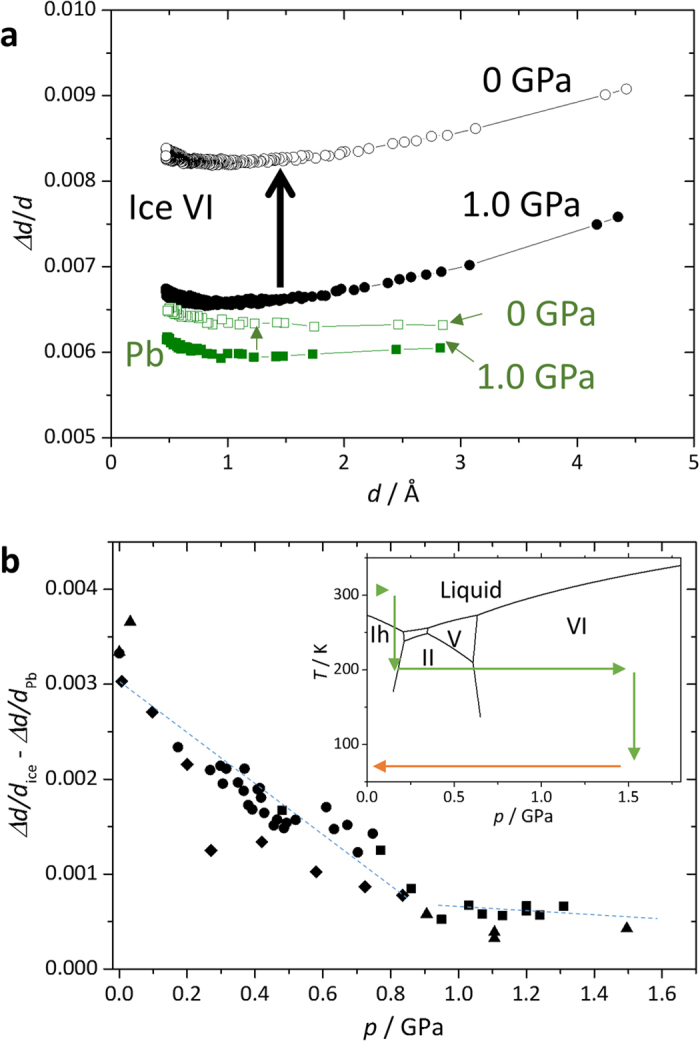
Peak broadening with decreasing pressure at 80 K. (**a**) Full width at half maximum divided by *d*-spacing (Δ*d*/*d*) as a function of *d*-spacing for ice VI (circle) and lead (square). Filled symbols show the data before decompression at 1.0 GPa, whereas open symbols correspond to 0 GPa after decompression. (**b**) Δ*d*/*d* for the most intense peak of ice VI with that of lead subtracted with decreasing pressure. The corresponding *p*–*T* path is denoted as an orange arrow in the phase diagram in the inset. Different symbols denote different runs. Dotted lines show guides for eyes.

In addition to the clear finding of additional peaks or *c*/*a* increase, an interesting feature of the Bragg peak broadening was observed in ice VI when the samples were downloaded to ambient pressure at 80 K (path *e*). The Bragg peaks of lead, which were included in the sample room as a pressure marker, were not broadened as much as those of ice, and the Δ*d*/*d* of ice constantly increased with decreasing pressure (Fig. 3a). The peak broadening in a diffraction pattern is generally caused by two factors: insufficient crystalline size and/or microstrain in the crystal. The fact that Δ*d*/*d* constantly increased regardless the *d*-spacing shows that the peak broadening of ice could be caused by microstrain rather than small crystalline size^21^. Small crystalline size will affect so as to change Δ*d* constantly rather than Δ*d/d* constantly due to the small number of termination of the Laue function. These findings imply two possibilities. (i) The peak broadening is an intrinsic behavior of ice rather than an external deviatoric stress owing to downloading, or (ii) the peak broadening is caused by deviatoric stress, and only the difference of compressibility between ice and lead differentiates the broadness. As the actual behavior could not reflect just one of these two extremes but must be to some extent a mixture, we plotted (Δ*d/d*_ice_ -Δ*d/d*_Pb_) as a function of pressure in order to reduce the component caused by the external deviatoric stress (Fig. 3b). As shown in the plot, peak broadening occurred below ~0.9 GPa. Interestingly, peak broadening was also observed prior to the appearance of additional peaks or the increase of *c*/*a* when the ordering occurred at ambient pressure and the peaks also sharpened correspondingly while disordering at 0.45 GPa (Fig. 2b,d). All of these observations suggest that the peak broadening was caused by lattice mismatch induced by locally ordered domains in ice VI, which are not sufficiently large to form Bragg peaks. Therefore, the ice XV is likely more stable than ice VI at *p*–*T* conditions at 80 K and below 0.9 GPa and was kinetically unobserved over the experimental time scale. It is noteworthy that the peak broadening with decreasing pressure found in this study is similar to the phenomena observed in ice VIII^22^, known as ice VIII’, showing structural instability under lower pressure. This coincidence also suggests ice VI to be unstable at pressures below 0.9 GPa.

### Effect of dopant

The results of a comparative neutron diffraction experiment using a sample not doped with deuterium chloride (DCl) also showed an increase of the *c*/*a* ratio associated with hydrogen ordering while annealing at ambient pressure (Supplementary Fig. S4). The rate of increase of *c*/*a* was mostly identical for the doped and pure samples, except that the initial value of *c*/*a* at 84 K was slightly higher for the doped sample. This result demonstrates that DCl doping did not significantly enhance the hydrogen ordering phase transition in the case of this study. Our result showing that the ordering transition did not occur at high pressure contradicts those of a previous study^4^ in which ice XV was observed at 80 K and 0.9 GPa. Recent heat capacity measurements clearly verified that doped DCl facilitates hydrogen ordering^14^. The difference was probably caused by the relatively slow cooling rate in path *b* (i.e., the sample solution was cooled at ~10 K/min herein), whereas it may have been much faster in the previous studies, wherein the samples were directly soaked in liquid nitrogen, with the quick quench allowing DCl to be solved into D_2_O ice crystals as solid solutions. If the solubility of DCl into D_2_O ice Ih is on a similar order to that of HCl into H_2_O ice Ih, then it could be on the order of 10^−7^–10^−5^ mol/mol at equilibrium^23^, which is significantly lower than 0.01 mol/L (2 × 10^−4 ^mol/mol). Thus, in this study the DCl component would be expelled from ice crystals owing to the slower cooling rate. In other words, the effects of the relatively insignificant doping could be negligible.

### 45 completely ordered configurations

There are 45 symmetry-distinct configurations for completely ordered forms of ice VI (see Methods), when the unit cell size is unchanged for the transition to ice XV. Among the 45 configurations, there are 12 pairs having a common Patterson function or a symmetrically equivalent Patterson function (i.e., they are homometric structures in terms of the neutron powder diffraction method if the ordered forms have no positional displacement from the disordered form). Thus, the remaining 33 configurations are experimentally distinguishable by neutron powder diffraction methods (see Supplementary Table S1). The Landau theory for phase transition could reduce the possible space groups if the symmetry reduction is caused by a single irreducible representation^24^. This will be discussed later in more detail, but in this section we will look at all 45 configurations including the experimentally indistinguishable pairs in order to explicitly compare all possibilities.

**Figure 4:**
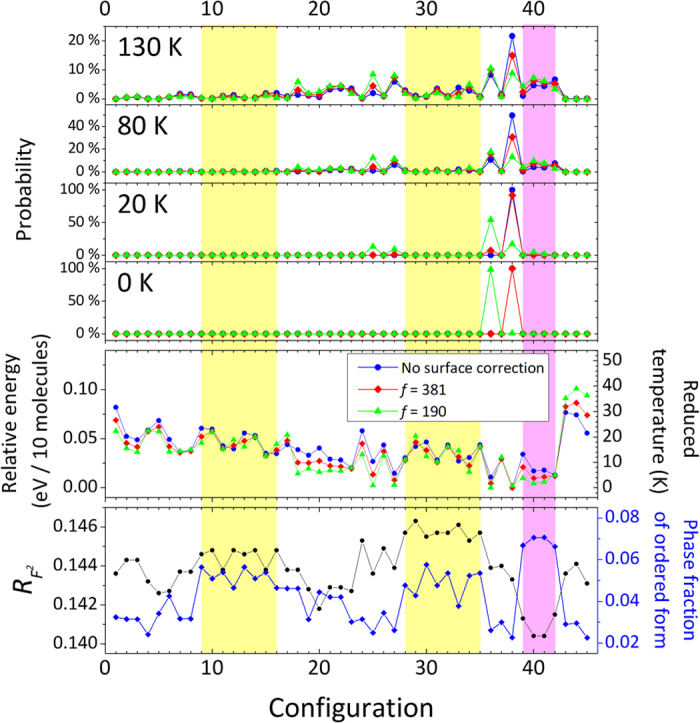
Results of Rietveld refinements and the DFT calculations for 45 completely ordered configurations. The Rietveld refinements were first conducted for the neutron diffraction pattern obtained at 80 K at ambient pressure after annealing at 128 K (see also Fig. 6) using the disordered ice VI structure model, and the ordered structure models were induced from the refined structure parameters. Then, the observed patterns were re-refined into two disordered and ordered phases by fixing all the parameters of both phases except the scale factors. Yellow and pink shaded areas show the configurations having *N*_*z*_ = 1 and 2, respectively (see equation (1) for the definition of *N*_*z*_). Relative energies per 10 molecules in a unit cell in eV (2^nd^ row from bottom), which are the differential energies with zero-point energy and surface corrections for *f* = 180 and 381 (see Methods for more details) to the minimum energy in the 45 configurations, were derived from the DFT calculations. The corresponding reduced temperatures (*T*_reduced_) calculated from the relative energies per atom (*ΔE*) using the equation (*ΔE* = *k*_B_*T*_reduced_) are shown on the right axis. The probabilities for each configuration derived from the relative energies at four different temperatures (130, 80, 20, and 0 K, from top) are calculated assuming a Boltzmann distribution based on the calculated energy (see Methods for more details).

Rietveld refinements based on a two-phase model involving a mixture of ice VI and an ordered phase in 45 configurations with fixed atomic coordinates and *U*_iso_ revealed that four configurations belonging to the space groups (number of configurations), 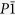 (39), *P*2_1_11 (40), *P*12_1_1 (41), and *P*11*n* (42) have slightly lower *R* factors and more phase fractions for the ordered phase (Fig. 4). Configurations 40 and 41 are homometric structures (i.e., they are experimentally indistinguishable owing to their symmetrically equivalent Patterson functions) and have identical *R* factors and phase fractions. Further refinements at varying atomic coordinates, *U*_iso_, etc., are technically possible but seem statistically meaningless because there would only be a few additionally observed Bragg peaks even though the number of parameters would increase by an order of magnitude over the disordered model refinement.

**Figure 5:**
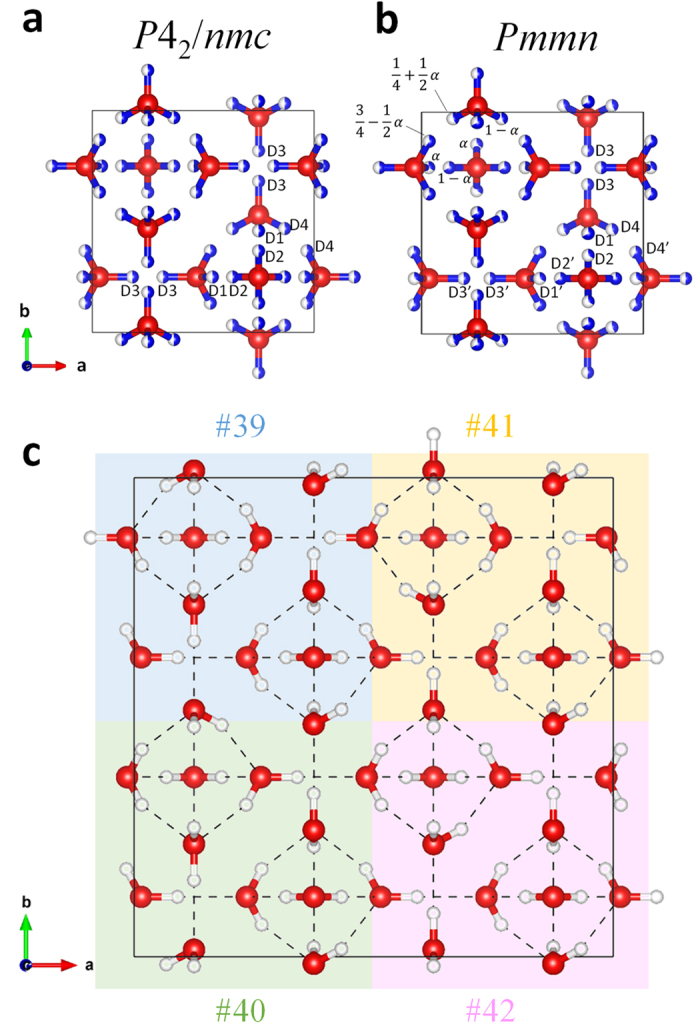
Structure models of ice VI and XV. (**a**) Crystal structure of ice VI. Labels for D atoms are based on Kuhs *et al*.^25^. (**b**) Partially ordered *Pmmn* structure model. Occupancies of D sites could be represented by a single parameter *α*, defined in ref. 9, as shown in left upper part of (**b**). Experimentally refined occupancies of D sites are denoted as the ratio of blue (occupied) and white (unoccupied) coloring of respective atoms. Detail structural parameters for the *Pmmn* model can be obtained from the cif file. (**c**) Four seamlessly adjacent ordered configurations (#39–#42) without breaking ice rules. Of the 45 symmetry distinct configurations, the four configurations shown here have the lowest *R* factors and the highest phase fractions obtained from Rietveld analysis of the neutron diffraction pattern.

Note that the calculated intensities for 003 reflection are significantly lower than those experimentally observed, even for the four configurations having lower *R*-factors (Fig. 4), and even though these configurations have the largest intensities of 003 reflection among the 45 configurations. When *l* is odd, contributions to the structure factors for 00*l* reflection from all oxygen atoms and D3 (see labels for D sites in Fig. 5) will be zero for the 45 configurations. Thus, other deuterium atoms, D1, D2, and D4 could contribute to the structure factor, which is proportional to a single parameter, *N*_*z*_, defined as follows:


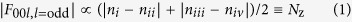


where *n*_i_–*n*_iv_ is the number of occupied deuterium atoms in D1, D2 or D4 sites, which are generated from the disordered model by means of four operations on the *z* coordinate: (i) *z*, (ii) *z *+1/2, (iii) −*z*, and (iv) −*z *+ 1/2. *N*_*z*_ will be the same, irrespective of which D1, D2 or D4 site is chosen. The 45 configurations can be classified into three groups in terms of the value of *N*_*z*_, as listed in Supplementary Table S1. Only four configurations (39–42) have *N*_*z*_ = 2, whereas other configurations have *N*_*z*_ = 0 or 1 (see Fig. 4), and therefore the four configurations have larger structure factors for 00*l* (*l* = odd) reflection. For most of the configurations except those from 43 to 45, *N*_*z*_ is equivalent to a number of “C-type” networks, one of three (A, B, and C) symmetrically distinct configurations for single H-bonding networks defined in previous works^4^,^13^,^14^ (see Fig. 3 in ref. 4). The appearance of 003 suggests that the C-type network is preferable to the A or B-type networks, as was mentioned in the previous neutron study^4^. Nevertheless, we emphasize here that configurations without the C-type network should not be excluded from possible ordered configurations as the only restriction of 003 reflection is that one, but not necessarily all, of the existing configurations should have *N*_*z*_ = 1 or 2. Further, note that the configurations from 43 to 45 have two C-type networks in their structures but have *N*_*z*_ = 0, so they do not contribute to the intensity of 003.

The DFT calculations for the 45 configurations revealed that the *Pc* (#38)^18^ configuration has the least energy, which is consistent with previous studies^11^,^12^,^17^. Conversely, Nanda and Beran^16^ predicted 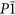 (#39) to be the most favored structure based on their calculations treating the long-range and polarization effect by applying fragment-based hybrid many-body interaction quantum mechanics/molecular mechanics methods. We also realized that, as indicated by Del-Ben *et al*., the surface correction term could be significant when a ferroelectric domain nucleates in a para-electric medium. The *Pc* (#38) configuration was particularly affected by the surface correction term (Fig. 4), and the *P*12_1_1 (#36) would have the least energy in cases wherein the surface correction term is relatively large (*f* = 180, see Methods for more details). Importantly, our calculations revealed that the relative energies for all the 45 configurations are within just 0.10 eV per 10 molecules, which corresponds with the reduced temperature of 39 K. As the energy difference between several favored configurations is even lower—within a few K—the least energy configuration may be altered depending on calculation method details and the surrounding conditions.

We would at least indicate that none of the single configurations has a particular dominance over others in terms of both the Rietveld refinements and the DFT calculations, but several configurations have similar likelihood at the temperatures we investigated. Our DFT calculations showed that several configurations may appear even at 20 K (Fig. 4). Thus, the observed ordered phase should be considered as a partially ordered structure, as will be discussed in next section, rather than a completely ordered one.

### Refinements on partially ordered *Pmmn* model

**Figure 6:**
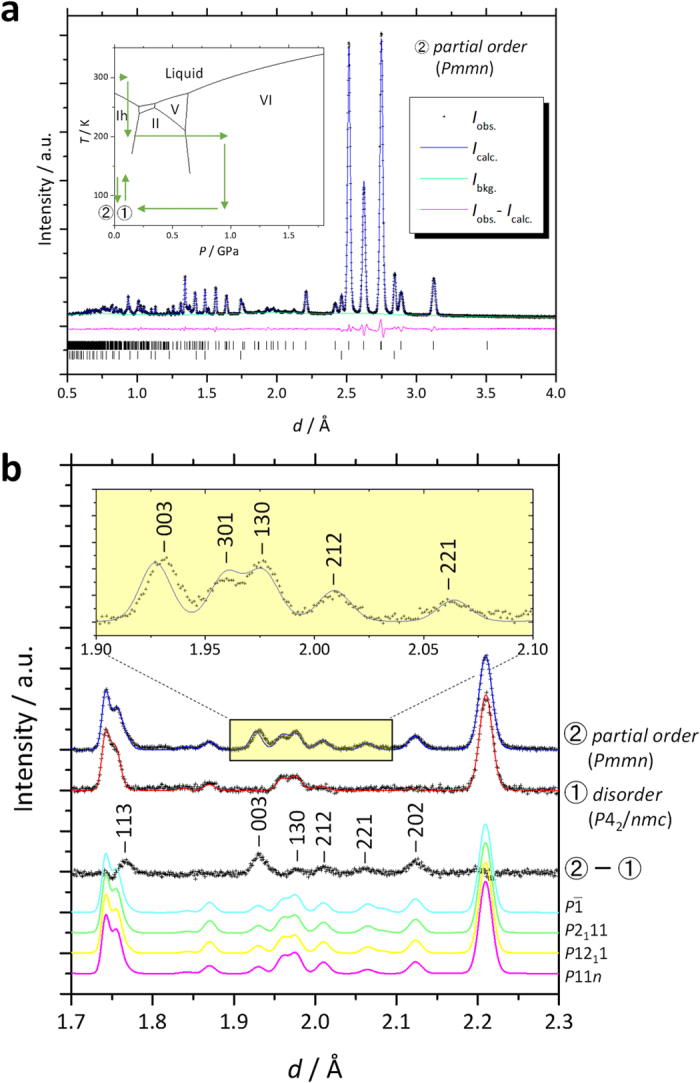
Results of structure refinement based on partially ordered model. (**a**) The results of the Rietveld refinement based on partially ordered *Pmmn* model for a neutron diffraction pattern taken at 80 K and ambient pressure after annealing at 130 K (denoted as ➁ in inset). (**b**) The diffraction pattern in the region where peaks from ordered form were observed, with a further enlarged area with the reflection indices shown as an inset. The diffraction pattern taken at the same *p*–*T* but just after recovery at ambient pressure (denoted in ➀ in the inset) is shown with the results of refinement based on a disordered *P*4_2_/*nmc* model. The difference between the two patterns with indices (➁−➀) and the calculated patterns for completely ordered models with space groups, 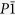 (#39), *P*2_1_11 (#40), *P*12_1_1 (#41), and *P*11n (#42), which have *N*_z_ = 2 and show a better fit to the observed pattern (see Fig. 4), are shown as references.

In the previous section, the completely ordered 45 configurations deduced from the disordered ice VI model were compared with the observed data. Here we inductively constrain the ordered model based on the observed diffraction pattern. The additionally appearing Bragg peaks included 003, 221, and 113 reflections (Fig. 6), which correspond to the extinction rules *hhl*:*l* = 2*n* and 00*l*:*l* = 2*n*, for the *P*4_2_/*nmc* space group of ice VI. On the other hand, the other reflection conditions of the space group, *hk*0:*h* + *k* = 2*n* and *h*00:*h* = 2*n*, are still fulfilled. It is also confirmed that the transition occurs without losing translational symmetry (i.e., zone center transition; **k** = 0, where **k** means wave vector), because all additional peaks can be indexed as integers. The obtained reflection conditions suggest *Pmmn* as the space group with the highest symmetry in the possible subgroups of *P*4_2_/*nmc* meeting the condition **k** = 0. Note that we could not exclude the possibility that lower symmetry space groups such as *Pm*2_1_*n* or *P*2_1_*mn* fulfil the conditions mentioned above. It should also be mentioned that the space group *Pmmn* has previously been proposed as an ordered form of ice VI as ice VI’ in the first neutron diffraction study by Kamb^6^. Although we could not directly compare to the patterns of ice VI’ and XV due to the missing the diffraction pattern of ice VI’ in ref. 6, the ice VI’ found by Kamb would be identical to ice XV, considering the fact that the ice VI’ formed at ambient pressure after downloading at low temperature like ice XV in this study.

The symmetry in the *Pmmn* space group and the ice rules constrain hydrogen occupancies such that the only single parameter *α*, as defined in Handa *et al*.^9^, is a free parameter for the occupancies of totally 7 D sites (Fig. 5b). The Rietveld refinement based on the partially ordered *Pmmn* model with varying atomic coordinates, *U*_iso_, and the parameter *α* converging stably yield *α* = 0.33 (Supplementary Tables S1 and S2). A structural comparison between disordered ice VI and the partially ordered ice XV based on the *Pmmn* model is shown in Supplementary Fig. S6, but we do not discuss it here in detail.

## Discussion

The partially ordered *Pmmn* model shows the best fit to the observed data among all the completely ordered models, including the four configurations with *N*_z_ = 2 (Fig. 6). In addition, the *Pmmn* space group does not conflict with Landau theory because it can be obtained from the parent space group *P*4_2_/*nmc* via a single irreducible representation, B_1g_. However, it should be mentioned that the constraint to the symmetry from Landau theory should be more carefully discussed because some preconditions for Landau theory are not met in this case. First, as the phase transition between ices VI and XV is not second order but weak first order, the rotations of water molecules do not occur simultaneously in the entire crystal but propagate through the crystal. In other words, the phase transition mechanism involves nucleation and growth and each nucleus does not have to result from the same irreducible representation. Second, as there are two H-bonding networks in ice VI/XV, it is not surprising that two or more order parameters are coupled and not only a single irreducible representation relates to the phase transition^14^. In other ice crystals, for example, the pairs of ice V (*A*2/*a*)–XIII (*P*2_1_/*a*) and ice XII (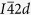)–XIV (*P*2_1_2_1_2_1_), the condition that space groups for lower symmetry phases are derived from single irreducible representations of the parent space groups is not actually obeyed. Although Salzmann *et al*.^4^ suggested that 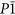 is the most plausible space group based on Landau theory, it should not be trivial.

Interstingly, *Pmmn* is a common supergroup of the space groups for the four configurations with *N*_z_ = 2 (i.e., 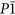 (#39), *P*2_1_11 (#40), *P*12_1_1 (#41), and *P*11*n* (#42)). We also realized that these four configurations could be adjacent each other without breaking ice rules (Fig. 5c). The DFT calculations for the mixed configuration with 2 × 2 × 1 unit cell as shown in Fig. 5c show that the enthalpy difference with respect to the averaged enthalpy of the four configurations is very small (*ΔH* = 0.000626 eV/10 molecule), thereby implying that ice XV comprises a mixture of micro domains, with each domain having locally ordered motifs. The existence of domain structures is supported by the observation that the position of 003 reflection, the only clearly observable 00*l* (*l* = odd) reflection, slightly deviates between calculated and observed patterns (see inset in Fig. 6b). Although the space group *Pmmn* itself suggests an antiferroelectrically ordered state because of the presence of a center of symmetry, this does not mean that the crystal physically possesses antiferroelectricity. Ferroelectricity may appear under just an electric field and its presence does not conflict with dielectric measurement^7^ when the locally ordered domain has configuration motifs without center of symmetry. Note that this scenario, which solves the disparity between neutron diffraction observations and dielectric measurements, has already been implied by Kamb^6^. The high degree of consistency of the partially ordered structure model to the observed neutron diffraction, the domain structure suggested by the deviation of the 003 peak, and the small energy differences among several favored configurations as revealed by DFT calculations, all support the Kamb’s scenario proposed more than 40 years ago. On the other hand, it could be experimentally difficult to obtain a single completely ordered configuration, which requires very low temperature. However, the rotation of water molecules would be too sluggish at these conditions unless an effective dopant is incorporated, as was recently done by doping HCl in the case of ice XII-XIV^26^, or unless another external field such as an electric field is applied to the sample. From the viewpoint that an observer may disturb the observed system, it would not be surprising if ice VI became really ferroelectrically ordered under the dielectric measurements in the previous study^7^. The possibility that an electric field may trigger another ordered state is also indicated in the case of the ordered phase of ice VII, which shows ferroelectricity according to calculations^27^. Furthermore, in the case of ice XI, a theoretical study indicated that the electric field induced by a surface charge caused by dopants at the grain boundary contributes to the stability of the ferroelectrically ordered *Cmc*2_1_ configuration, and the true ground state of pure ice should be nonpolar^28^. It could be a common feature that the external and/or dopant-induced electric field stabilizes other configurations. In other words, the order–disorder pairs in ice polymorphs are no longer one-to-one correspondent pairs but one-disorder to *n*-order correspondence, with *n* possible configurations. Such undiscovered configurations are potentially detectable by neutron diffraction under an electric field, and exploring them will provide new insights into ice physics.

In terms of nomenclature for ice polymorphs, we would finally suggest the term for any ordered form of ice VI as ice XV without using a new Roman number, even if another completely or partially ordered form of ice VI is observed under electric fields, using other dopants, and/or other *p*–*T* conditions. In addition, a notation referencing the space group may be added in parenthesis, e.g., as “ice XV (*Pmmn*)”, to differentiate from each other.

## Methods

### Neutron diffraction

Liquid DCl-doped D_2_O (0.01 mol/L) made from pure D_2_O (99.9%, Wako) and DCl solution (D 99.5%, 20 wt.% solution in D_2_O, Sigma Aldrich) and a piece of lead (0.03–0.05 g) as a pressure calibrant were loaded into encapsulating TiZr null metal gaskets sandwiched by a pair of tungsten carbide or alumina-toughened-zirconia anvils^29^ and loaded by the Mito system^20^, which is an opposed type hydraulic press with flow paths for liquid nitrogen to control temperature. A comparative experiment for a sample without DCl doping was also conducted. A typical *p*–*T* path is drawn in Fig. 1, with actual *p–T* paths were dependent on the respective runs, which are described in detail with the respective results in the Results section. Neutron diffraction patterns were measured on the BL11 beamline (PLANET)^19^ in the Material and Life Science Experiment Facility (MLF) of J-PARC, Ibaraki, Japan. The sample pressure was estimated from the observed lattice parameter of lead using its equation-of-states^30^, and the sample temperature was measured by two K-type thermocouples (TCs), which were calibrated in advance by a Pt conductive thermometer attached to the anvils (typical deviations of the two TCs were within 0.5 K, and no more than 2.0 K). More detailed procedures for sample setup, *p*–*T* control, data reduction, and refinements are described elsewhere^20^,^31^.

### DFT calculations

Quantum Espresso^32^ was used for the DFT calculations^33^,^34^. Quantum Espresso is a package for the calculation of electronic structure properties using a plain-wave basis set and pseudopotentials. We used Perdew–Burke–Ernzerhof (so called PBE) type nonempirical exchange-correlation functionals^35^ for this study, which give similar energy tendency^17^ to approximations from wave function theory such as a MP2^36^ and RPA^37^. The pseudopotentials were derived using the Troullier–Martins Method^38^. The dispersion effects were not taken into account in this study, because it does not significantly alter the relative energy^17^. The enthalpies of 45 possible configurations for ice XV were calculated within a unit cell with a kinetic energy cutoff of 70 Ry and a Brillouin zone k mesh of 5 × 5 × 6. The cell and atomic parameters were optimized using BFGS quasi-Newtonian methods^39^,^40^,^41^,^42^ at atmospheric pressure. The phonons were calculated using the density functional perturbation theory^43^. The effects of zero-point motions were corrected by adding the phonon energy (*U*_ZP_ = 1/2 Σ_*n*_*ħω*_n_) to the enthalpy obtained from the DFT calculations. An additional surface correction term, which was discussed in detail in previous studies^44^,^45^,^46^,^47^, was posteriorly considered as proposed by Del Ben *et al*.^17^:


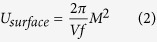


where *M* is the dipole moment, *V* is the simulation cell volume, and *f* is (2ε+1) when the system is embedded in a spherical cavity of a medium of dielectric constant, ε. The values *f* = 391 and 180 were chosen, which were the same as in the previous study^17^. The probability of occurrence of the *i*th configuration (*p*_*i*_), assuming the Boltzmann distribution on the calculated energy (*E*_*i*_), is given by^48^


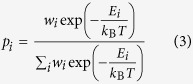


where *w*_*i*_ is number of the symmetry equivalent configurations (see Supplementary Tables S3 and S4).

### Generation of possible ordered configurations

The possible ordered configurations for ice XV were derived in previous studies^11^,^12^ by using the concept of a graph invariant, which is a parameter that is invariant to symmetry operation on a parent phase. Graph invariants may be useful in estimating physical properties of larger cells once the property is evaluated for a unit cell (see the review paper by Singer and Knight)^49^. However, the decision on how the graph invariants should be chosen to distinguish symmetrically identical configurations is not trivial. For example, the first- and second-order graph invariants defined in ref. 50. can only classify the 33 configurations having the common Patterson function or its symmetrically equivalents, as shown in Results section. It is necessary to consider another graph invariant to distinguish configurations that are not symmetry equivalent. Here we describe a straightforward way to generate the possible ordered configurations satisfying ice rules.

The structure of ice VI consists of two hydrogen-bonding (H-bonding) networks. The two networks are isolated from each other in terms of H-bond and each network has a unit comprising five water molecules (called “isomers” hereafter) as labeled 1–5 in Supplementary Fig. S5a. Since all the molecules in an isomer are bound to the other four molecules (Supplementary Fig. S5b), the H-bonding network for a single isomer can be simplified as shown in Supplementary Fig. S5c, which is coincidently well known in the graph theory as complete graph K5. An ordered configuration can correspond to a directed graph (Supplementary Fig. S5d,e) in which an arrow shows the direction from donor to acceptor in an H-bond. If any points (called vertices in graph theory) in a graph have four arrows with two-in and two-out directions, the corresponding H-bonding network obeys the ice rules. All possible directed graphs obeying the ice rules can be derived by permutation of the vertices while fixing the directions of the edges because all vertices are identical in the complete graph. One of the five vertices of the graph should be fixed in the permutation to avoid over-counting, yielding (5–1)! = 24 allowed patterns. Because two H-bond networks are isolated from each other, 24^2^ = 576 patterns can be considered as possible ordered configurations for a 1 × 1 × 1 unit cell. The number of possible configurations is even higher for larger unit cells, but as our experimental results can restrict the unit cell of ice XV to be identical to that of disordered ice VI (*i.e.*, the so-called Brillouin zone center transition, Results section) we did deal with larger unit cells in this study. Most of the 576 configurations are symmetrically identical to others, so we need to consider only symmetrically distinct configurations. If an ordered configuration (for instance, designated H_1_) is symmetrically equivalent to another (H_2_), H_1_ and H_2_ belong to the same space group (H), which should be a subgroup of the space group of ice VI (*P*4_2_/*nmc*, G). The operation (*g*) to convert H_1_ to H_2_ should be one of the 16 symmetry operations of G, and *g* should not belong to H:


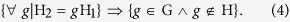


In other words, any symmetry operation *g* in G can convert an *i*^th^ configuration H_*i*_ to a nonidentical *j*^th^ configuration H_*j*_ or to H_*i*_ itself if *g* also belongs to H:


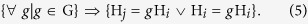



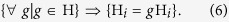


Because symmetry operations are distance preserving and bijective, any configuration converted by *g* from a configuration obeying the ice rules will also obey the ice rules. As a result, 576 configurations can be converted by 16 symmetry operations to other configurations (or themselves) within the 576, and this screening gives a full list of symmetry relations between the respective configurations. Finally, 45 symmetry distinct configurations, which are explicitly tabulated in Supplementary Table S3, are found in the list.

Among the 16 symmetry operations in G, eight operations do not exchange two H-bonding networks, whereas the other eight do. Thus, if we focus on the symmetry of a single H-bonding network, each configuration has eight symmetrically equivalents, yielding three (=24/8) configurations for a single network that could be chosen as symmetrically inequivalent configurations (*i.e.*, these regarded as “A-”, “B-”, and “C-type” networks in previous works)^4^,^13^,^14^,^15^.

## Additional Information

**How to cite this article**: Komatsu, K. *et al*. Partially ordered state of ice XV. *Sci. Rep.* **6**, 28920; doi: 10.1038/srep28920 (2016).

## Supplementary Material

Supplementary Information
