# Erratum: Partially ordered state of ice XV

**DOI:** 10.1038/srep30994

**Published:** 2016-08-12

**Authors:** K. Komatsu, F. Noritake, S. Machida, A. Sano-Furukawa, T. Hattori, R. Yamane, H. Kagi

Scientific Reports 6: Article number: 28920; 10.1038/srep28920 published online: 07 04 2016; updated: 08 12 2016.

The original version of this Article incorrectly listed ‘School of Material and Chemical Technology, Tokyo Institute of Technology, 2-12-1, Ookayama, Meguro-Ku, Tokyo 152-8550, Japan’ as a present address for K. Komatsu. This error has now been corrected in the HTML and PDF versions of the Article.

