# Supplementary material for: Partially ordered state of ice XV
Source: Sci Rep. 2016 Jul 4;6:28920. doi: 10.1038/srep28920 (PMC4931510; doi:10.1038/srep28920)
Supplement: Supplementary Information [file srep28920-s1.pdf]

Supplementary Information

Partially ordered state of ice XV

K. Komatsu,[1,a)] F. Noritake,[1] S. Machida,[2] A. Sano-Furukawa,[3] T. Hattori,[3] R. Yamane,[1] H. Kagi,[1]

[1]*Geochemical Research Center, Graduate School of Science, The University of Tokyo, Hongo 7-3-1, Bunkyo-ku, Tokyo 113-0033, Japan*

[2]*CROSS-Tokai, Research Center for Neutron Science and Technology, IQBRC Bldg, 162-1 Shirakata, Tokai, Ibaraki 319-1106, Japan*

[3]*J-PARC Center, Japan Atomic Energy Agency, 2-4 Shirakata-Shirane, Tokai, Ibaraki 319-1195, Japan*

---

a) Corresponding author. E-mail: kom@eqchem.s.u-tokyo.ac.jp

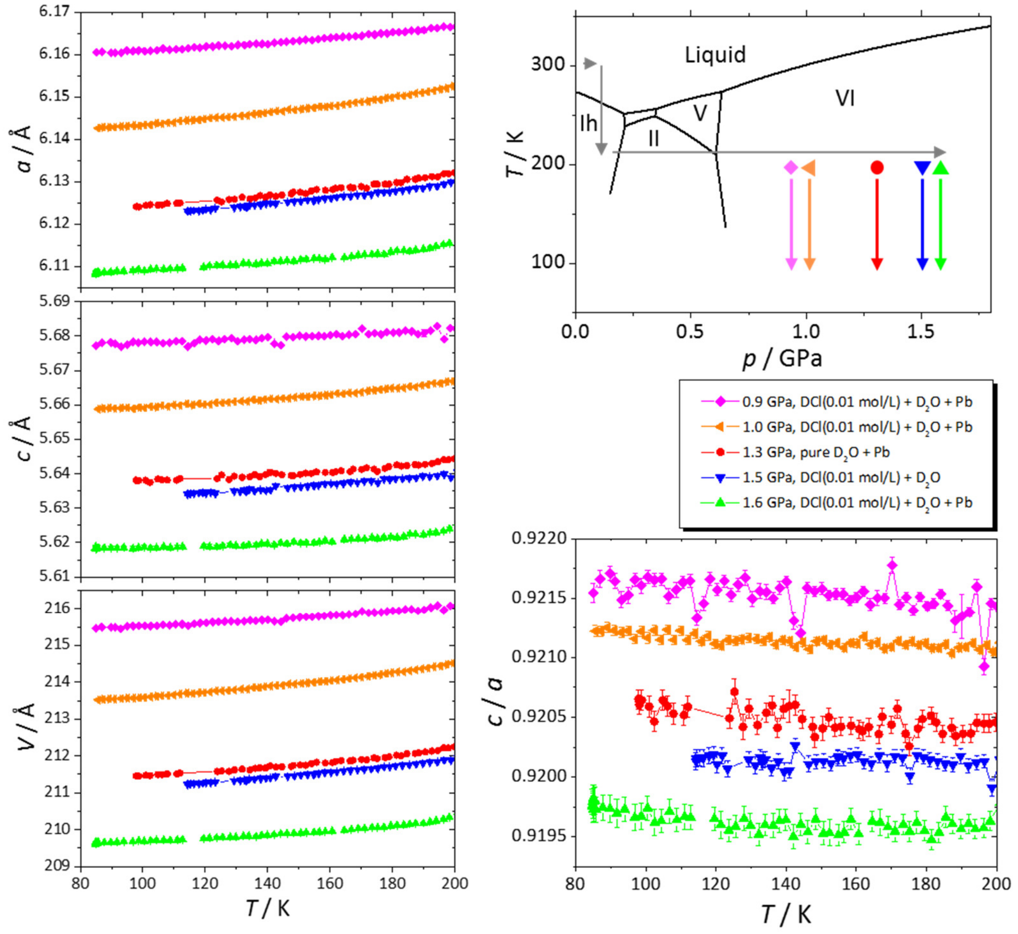

Supplementary Figure S1 | Lattice parameters of ice VI with decreasing temperature under pressure. Different symbols/colors show the results for different pressures over 5 runs, as indicated by the corresponding color's arrows in the upper right phase diagram of ice. Samples are DCl doped D_2O (0.01 mol/L) except for the data taken at 1.3 GPa (Red circle), which is from a pure D_2O sample.

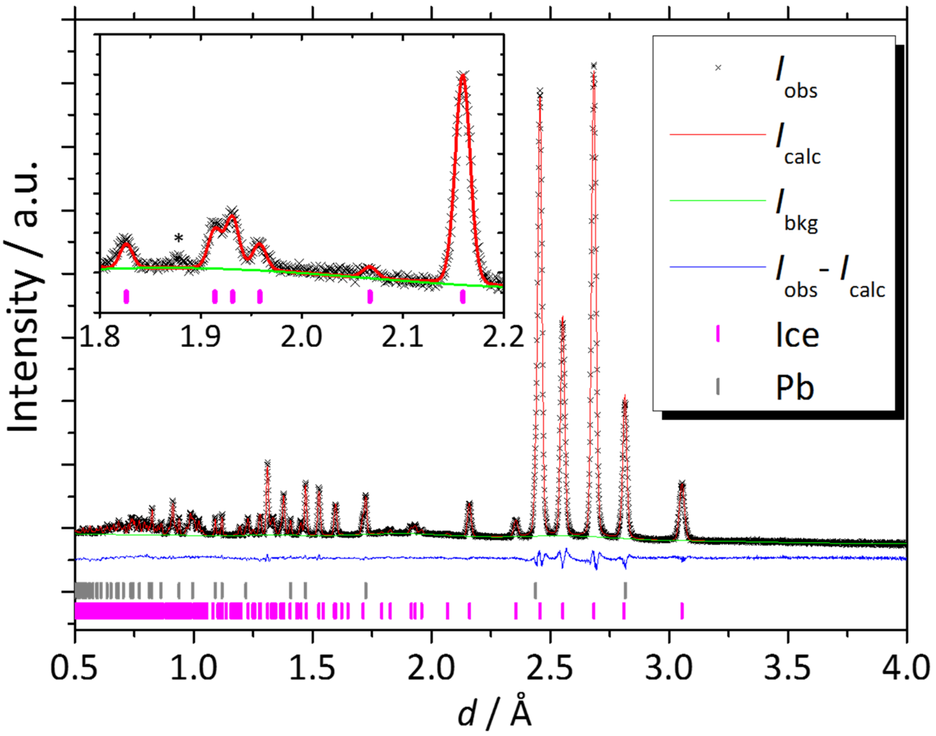

Supplementary Figure S2| Result of Rietveld refinement of disordered ice VI. The neutron diffraction pattern was taken at 1.6 GPa and 85 K after cooling from 200 K under pressure (shown by green line in Supplementary Fig. S1). Inset shows the enlarged region where peaks from ordered form are expected to be observed. All peaks in the pattern can be indexed as ice VI (pink tick marks) with disordered model or lead pressure marker (gray tick marks), and only one visible peak (asterisk in the inset) was derived from tungsten carbide used as anvils.

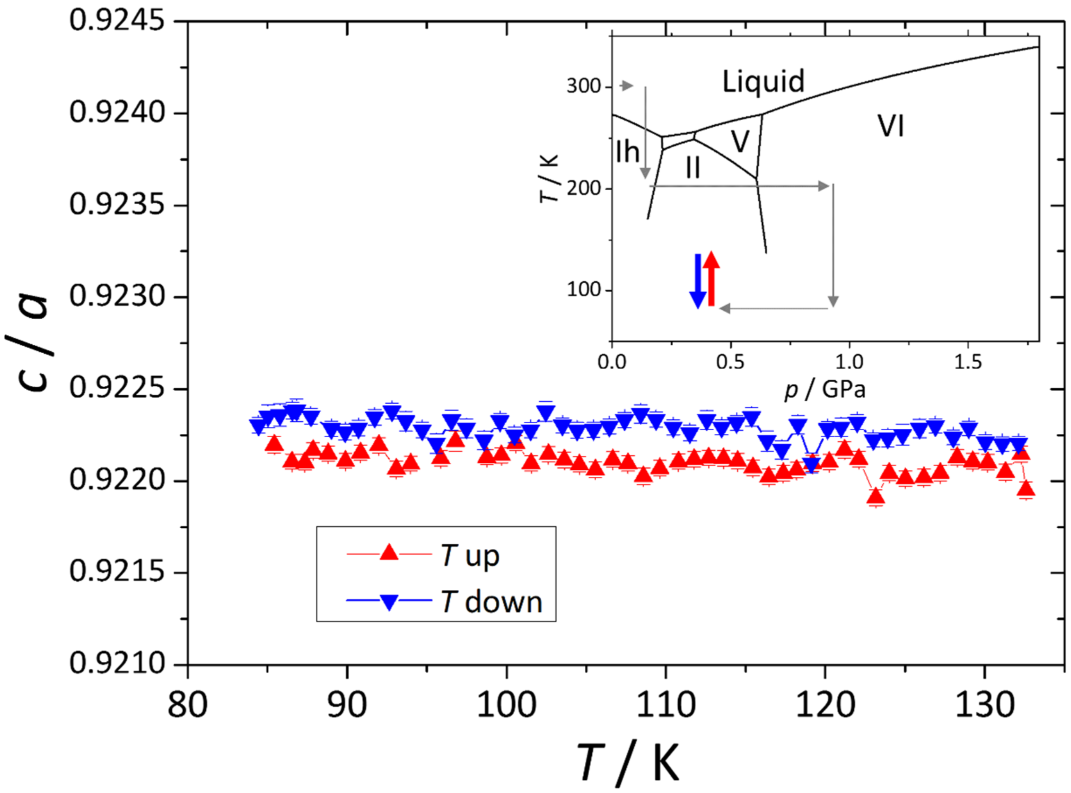

Supplementary Figure S3| Axial ratios *c*/*a* of ice VI with increasing (red) and decreasing (blue) temperature at ~0.4 GPa. Inset shows the *p*–*T* paths over which the axial ratios were obtained on the phase diagram of ice. The axial ratio did not change with temperature, unlike in Fig. 2, showing that hydrogen ordering did not occur under these conditions.

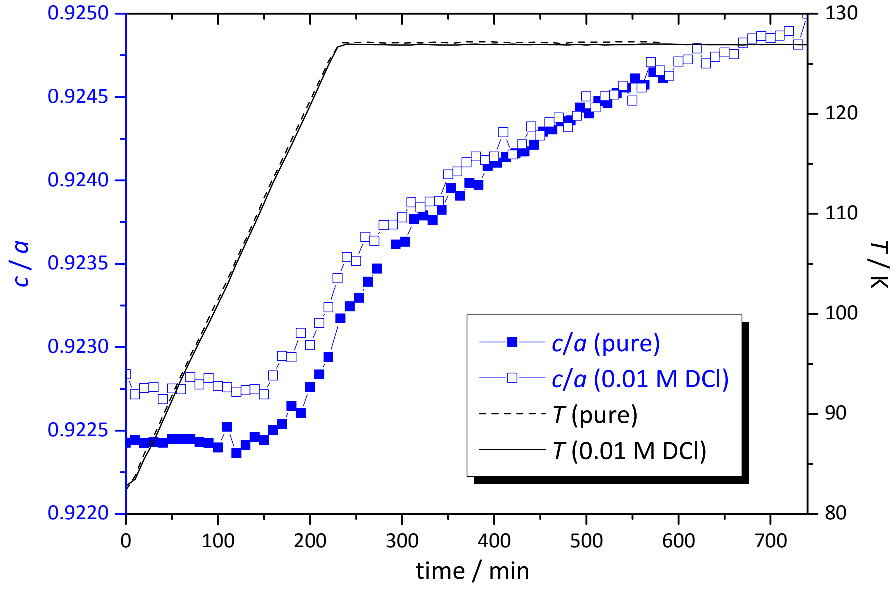

Supplementary Figure S4 | Comparison of axial ratio between pure and DCl doped samples. The axial ratio (*c/a*) for the doped (0.01 mol/L DCl) sample is the same as shown in Fig. 2, and the same procedures to collect diffraction patterns were applied for the pure sample. Averaged temperatures measured by two K-type thermocouples are also shown for both pure (dotted line) and doped (normal line) samples, showing high correspondence.

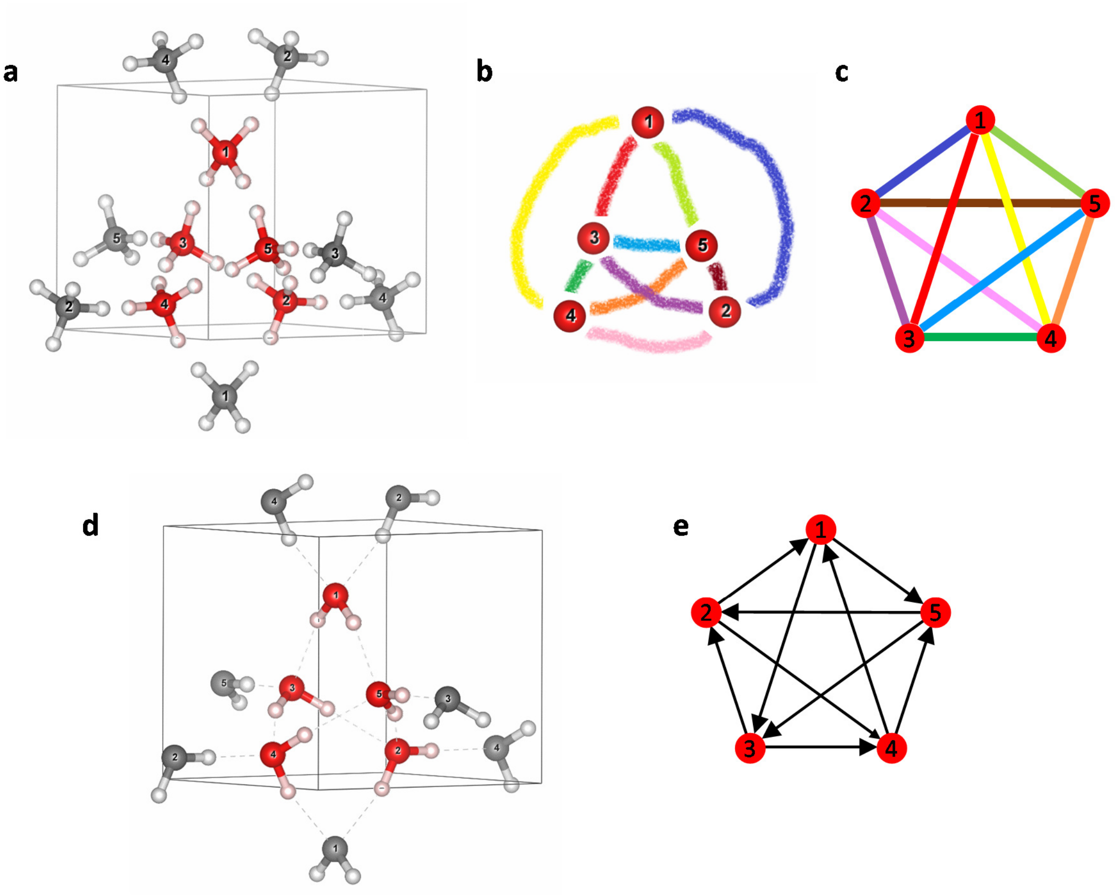

Supplementary Figure S5 | Structure of H-bonding network in ice VI and its ordered form. (a) A single H-bonding network. Five colored molecules labeled 1–5 comprise an isomer, considered to be the periodic unit of the single H-bonding network. (b) H-bonding network schematically represented. (c) Complete graph K5. Colors of edges correspond to those in (b). (d) An example of ordered configuration of ice VI obeying the ice rules, and (e) its graphical representation by directed graph. See text for more details.

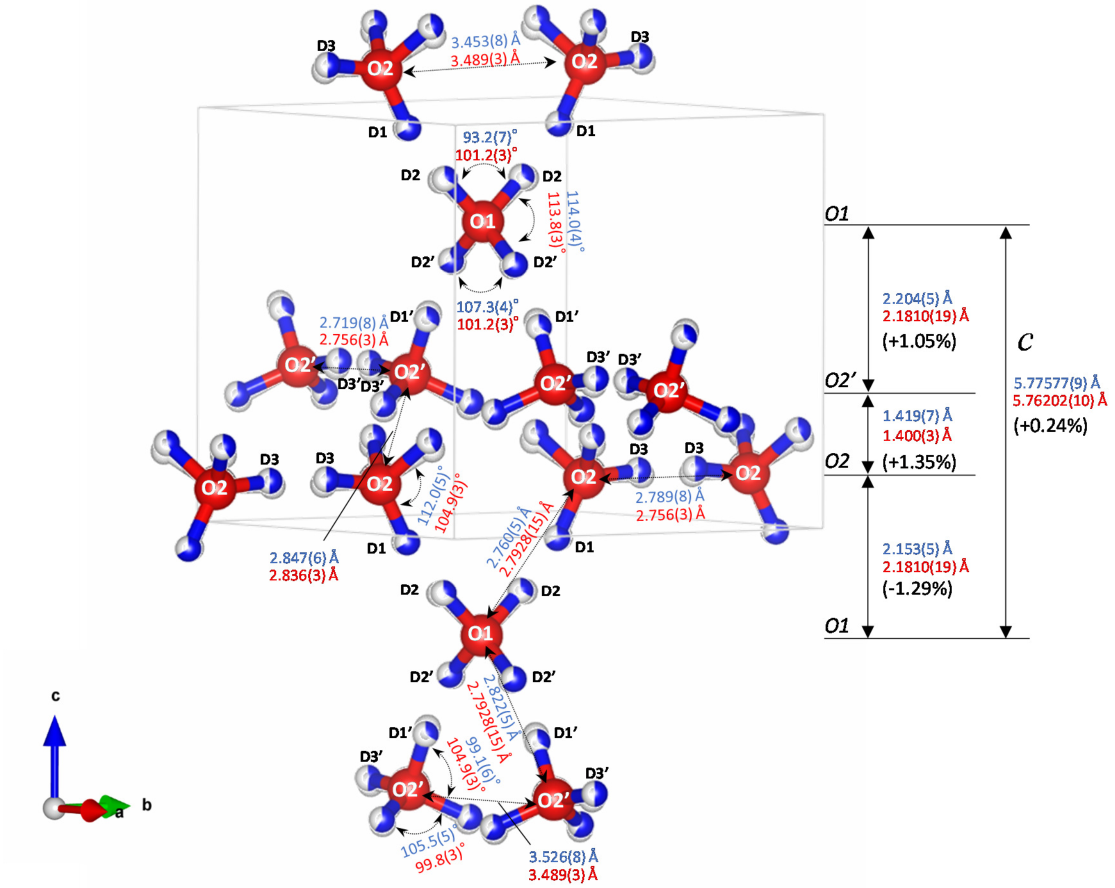

Supplementary Figure S6 | Structure comparison between ice VI and XV. Structure of ice XV, with colors for a single hydrogen bonding network, is superimposed on the corresponding structure of ice VI in gray scale. Distances or angles with red and blue characters show values for ice VI and ice XV, respectively. Distances between oxygen planes along the *c*-axis, O1– O2', O2'– O2, and O2– O1 are also shown beside the structure. Elongation of the *c*-axis by ordering is dominant on the planes between O1–O2' and O2'–O2, whereas the distance between O2–O1 planes was shortened by ordering. Detailed structure parameters are given in Supplementary Tables S1 and S2 and also deposited in the Cambridge Crystallographic Data Centre (CCDC #1463847 and #1463849).

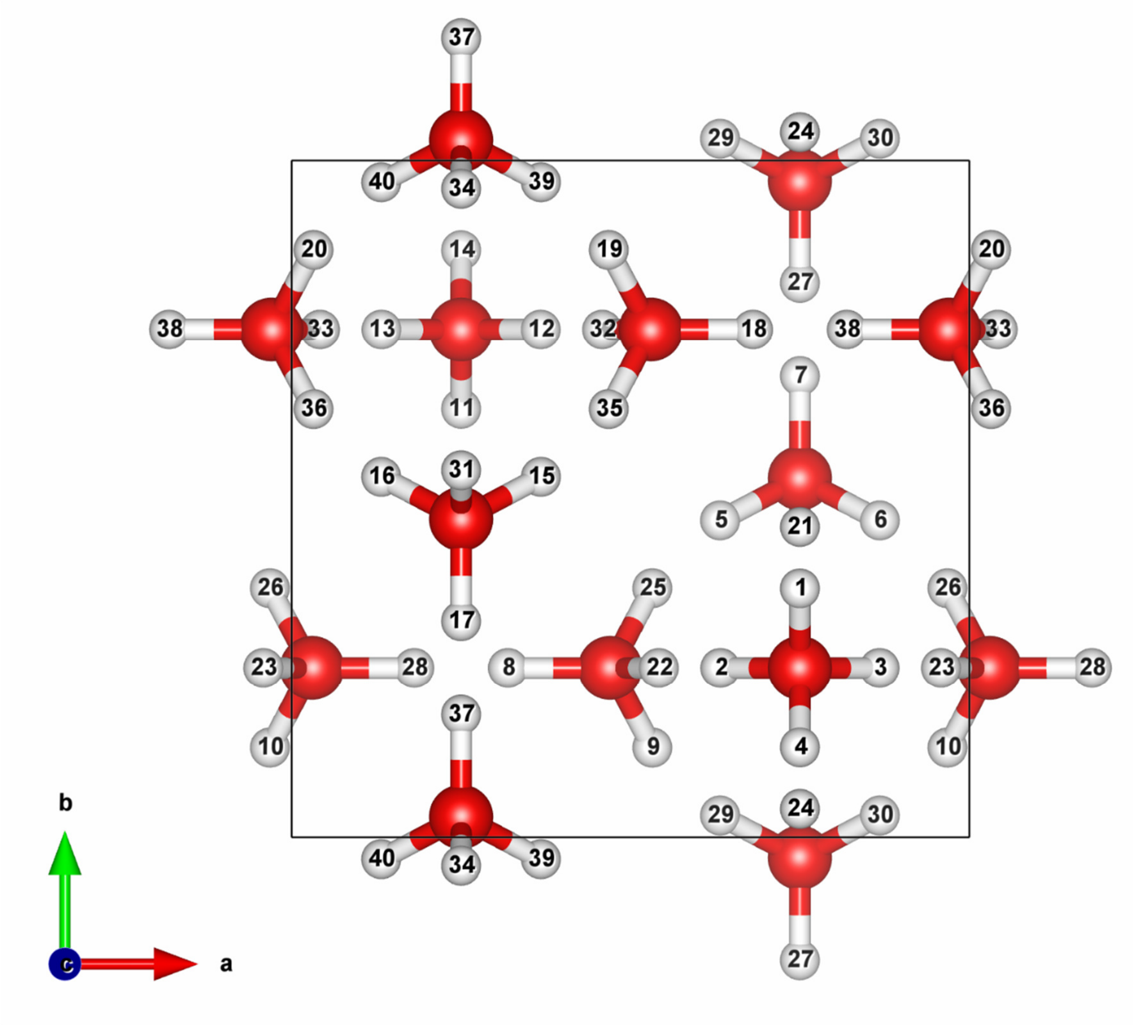

Supplementary Figure S7 | Crystal structure of ice VI. H site labels correspond to Supplementary Table S3.

Supplementary Table S1 | Experimental details of structure refinements for ice VI and XV.

Crystal data		
Phase name	ice VI	ice XV
Chemical formula	D_2O	D_2O
M_r	200.276	200.276
Crystal system, space group	$P4_2/nmc$	*Pmmn*
Temperature (K)*	84	84
Pressure (GPa)*	0.0001	0.0001
a (Å)	6.24460(6)	6.24495(6)
b (Å)	$= a$	6.24197(6)
c (Å)	5.76203(10)	5.77577(9)
V (Å^3)	224.691(4)	225.144(4)
Z	10	10
Radiation type	Spallation neutron	
Specimen shape, size (mm)	Irregular, 3.6 × 3.6 × 3.8	
Data collection		
Diffractometer	PLANET (BL11), MLF, J-PARC	
Specimen mounting	Pressure-temperature controlling system (the MITO system)	
Data collection mode	Transmission	
Scan method	Time-of-flight	
2θ values (°)	$2\theta_{min}$ = 89°, $2\theta_{max}$ = 101°	
Refinement		
R factors and goodness of fit		
R_p	0.0339	0.0298
R_{wp}	0.0389	0.0345
$R(F^2)$	0.1599	0.0918
χ^2	2.707	8.209
No. of data points	3525	3525
No. of parameters	26	30

Computer program: GSAS (Larson & Von Dreele, 2004)

*See text for detailed p-T paths.

Supplementary Table S2 | Atomic fractional coordinates and atomic displacement parameters for ice VI and XV.

Ice VI

Atom	x	y	z	U_{iso}	Occ.
O1	0.75	0.25	0.75	1.24(9)	1
O2	0.75	0.5293(3)	0.1285(3)	1.13(3)	1
D1	0.75	0.4604(6)	–0.0179(7)	1.88(7)	0.5
D2	0.75	0.3654(4)	–0.1472(6)	1.99(7)	0.5
D3	0.75	0.6806(4)	0.1258(4)	1.63(7)	0.5
D4	0.1343(3)	0.5324(4)	0.7899(4)	2.21(6)	0.5

Ice XV

Atom	x	y	z	U_{iso}	Occ.*
O1	0.75	0.25	0.75	1.22(3)	1
O2	0.75	0.5266(8)	0.1228(7)	1.22(3)	1
O2'	0.5323(8)	0.75	0.6316(8)	1.22(3)	1
D1	0.75	0.4535(9)	–0.0192(11)	1.89(3)	$1 - \alpha$
D1'	0.468(2)	0.75	0.467(2)	1.89(3)	α
D2	0.75	0.3542(15)	–0.144(2)	1.89(3)	α
D2'	0.3743(8)	0.75	0.3488(12)	1.89(3)	$1 - \alpha$
D3	0.75	0.6731(10)	0.1383(10)	1.89(3)	0.5
D3'	0.6904(10)	0.75	0.6110(10)	1.89(3)	0.5
D4	0.1401(11)	0.5297(12)	0.7699(9)	1.89(3)	$1/4 + 1/2\ \alpha$
D4'	0.4661(10)	0.8685(8)	0.6974(7)	1.89(3)	$1/4 - 1/2\ \alpha$

*α = 0.334(4)

Supplementary Table S3 | 45 symmetry distinct configurations for completely ordered form of ice VI. Site labels correspond to those illustrated in Supplementary Fig. S7. Site occupancies for the ith site (s_i) for sites 21–40 can be obtained from those for sites 1–20 shown in this table by $s_i(21 \leq i \leq 40) = 1 - s_{i-20}$. S.G. (#): Space group and its number in International Tables for Crystallography. Note that the settings of space groups are chosen so as not to change the atomic fractional coordinates of ice VI. # of eq.: Number of equivalent configurations in 576 possible configurations which obey ice rules for 1 × 1 × 1 unit cell. E.I.: Experimentally indistinguishable configuration having the same powder neutron diffraction pattern. N_z: A parameter defined by eq. (1), which is proportional to F_{00l} (l = odd) (see text for more details). NB: Corresponding notation by Nanda and Beran[1].

config.	Site label 1	2	3	4	5	6	7	8	9	10	11	12	13	14	15	16	17	18	19	20	S.G. (#)	# of eq.	E.I.	N_z	NB
1	0	0	1	1	0	0	1	0	0	0	0	0	1	1	0	0	1	0	0	0	P-1 (2)	8		0	2B
2	0	0	1	1	0	0	1	0	0	0	0	0	1	1	0	1	0	0	0	1		16	3	0	
3	0	0	1	1	0	0	1	0	0	0	0	0	1	1	1	0	0	0	1	0		16	2	0	
4	0	0	1	1	0	0	1	0	0	0	0	0	1	1	1	0	0	1	0	1	P_c* (7)	8		0	9B1
5	0	0	1	1	0	0	1	0	0	0	0	1	0	1	0	0	1	1	0	0	$P2_111$ (4)	8		0	4B2
6	0	0	1	1	0	0	1	0	0	0	0	1	0	1	0	1	0	0	1	0		16		0	
7	0	0	1	1	0	0	1	0	0	0	0	1	0	1	0	1	0	1	0	1		16	8	0	
8	0	0	1	1	0	0	1	0	0	0	0	1	0	1	1	0	0	1	1	0		16	7	0	
9	0	0	1	1	0	0	1	0	0	0	0	1	1	0	0	0	1	0	1	0		16	13	1	
10	0	0	1	1	0	0	1	0	0	0	0	1	1	0	0	0	1	1	0	1		16	14	1	
11	0	0	1	1	0	0	1	0	0	0	0	1	1	0	0	1	0	0	1	1		16	15	1	
12	0	0	1	1	0	0	1	0	0	0	0	1	1	0	1	0	0	1	1	1		16	16	1	
13	0	0	1	1	0	0	1	0	0	0	1	0	0	1	0	1	1	0	0	0		16	9	1	
14	0	0	1	1	0	0	1	0	0	0	1	0	0	1	1	0	1	1	0	0		16	10	1	
15	0	0	1	1	0	0	1	0	0	0	1	0	0	1	1	1	0	0	1	0		16	11	1	
16	0	0	1	1	0	0	1	0	0	0	1	0	0	1	1	1	0	1	0	1		16	12	1	
17	0	0	1	1	0	0	1	0	0	0	1	0	1	0	0	1	1	0	0	1		16	18	0	
18	0	0	1	1	0	0	1	0	0	0	1	0	1	0	1	0	1	0	1	0		16	17	0	
19	0	0	1	1	0	0	1	0	0	0	1	0	1	0	1	1	0	0	1	1	$P12_11$ (4)	8		0	4B1
20	0	0	1	1	0	0	1	0	0	0	1	1	0	0	0	1	1	0	1	0	P_c** (7)	8		0	9B2
21	0	0	1	1	0	0	1	0	0	0	1	1	0	0	0	1	1	1	0	1		16	22	0	
22	0	0	1	1	0	0	1	0	0	0	1	1	0	0	1	0	1	1	1	0		16	21	0	
23	0	0	1	1	0	0	1	0	0	0	1	1	0	0	1	1	0	1	1	1	$P11n$ (7)	8		0	7B
24	0	0	1	1	0	1	0	0	0	1	0	0	1	1	0	1	0	0	0	1	P-1 (2)	8		0	2A
25	0	0	1	1	0	1	0	0	0	1	0	0	1	1	1	0	0	0	1	0	P_c* (7)	8		0	9A1
26	0	0	1	1	0	1	0	0	0	1	0	1	0	1	0	1	0	1	0	1		16		0	
27	0	0	1	1	0	1	0	0	0	1	0	1	0	1	1	0	0	1	1	0	$P2_111$ (4)	8	36	0	4A1
28	0	0	1	1	0	1	0	0	0	1	0	1	1	0	0	0	1	0	1	0		16	31	1	
29	0	0	1	1	0	1	0	0	0	1	0	1	1	0	0	0	1	1	0	1		16		1	
30	0	0	1	1	0	1	0	0	0	1	0	1	1	0	0	1	0	0	1	1		16		1	
31	0	0	1	1	0	1	0	0	0	1	0	1	1	0	1	0	0	1	1	1		16	28	1	
32	0	0	1	1	0	1	0	0	0	1	1	0	0	1	0	1	1	0	0	0		16	35	1	
33	0	0	1	1	0	1	0	0	0	1	1	0	0	1	1	0	1	1	0	0		16		1	
34	0	0	1	1	0	1	0	0	0	1	1	0	0	1	1	1	0	0	1	0		16		1	
35	0	0	1	1	0	1	0	0	0	1	1	0	0	1	1	1	0	1	0	1		16	32	1	
36	0	0	1	1	0	1	0	0	0	1	1	0	1	0	1	0	1	0	1	0	$P12_11$ (4)	8	27	0	4A2
37	0	0	1	1	0	1	0	0	0	1	1	1	0	0	0	1	1	1	0	1	$P11n$ (7)	8		0	7A
38	0	0	1	1	0	1	0	0	0	1	1	1	0	0	1	0	1	1	1	0	P_c** (7)	8		0	9A2
39	0	1	1	0	0	0	1	0	1	0	0	1	1	0	0	0	1	0	1	0	P-1 (2)	8		2	2C
40	0	1	1	0	0	0	1	0	1	0	0	1	1	0	0	0	1	1	0	1	$P2_111$ (4)	8	41	2	4C1
41	0	1	1	0	0	0	1	0	1	0	0	1	1	0	0	1	0	0	1	1	$P12_11$ (4)	8	40	2	4C2
42	0	1	1	0	0	0	1	0	1	0	0	1	1	0	1	0	0	1	1	1	$P11n$ (7)	8		2	7C
43	0	1	1	0	0	0	1	0	1	0	1	0	0	1	0	1	1	0	0	0	P_c** (7)	8		0	9C1
44	0	1	1	0	0	0	1	0	1	0	1	0	0	1	1	0	1	1	0	0		16		0	
45	0	1	1	0	0	0	1	0	1	0	1	0	0	1	1	1	0	1	0	1	P_c* (7)	8		0	9C2

*Basis vectors are transformed from the basis of 1st setting of P_c by (**a' b' c'**) = (**a** − **b a** + **b c**) so as not to change the atomic fractional coordinates.

Similarly, basis vectors are transformed by (a' b' c'**) = (**a** + **b** –**a** + **b c**) with an origin shift of $\frac{3}{4}\mathbf{a} + \frac{3}{4}\mathbf{b}$.

Supplementary Table S4 | Absolute energies and optimized lattice parameters for 45 symmetry distinct configurations from the DFT calculations. The surface correction terms (U_{surface}) based on f = 381 and 190 (see Methods) and the zero-point motion correction (U_{ZP}) are also shown.

	Quantum Espresso PBE energy (eV/10 molecules)				Lattice parameter (Å, °)					
config.	No correction	f = 381	f = 190	U_{ZP}	a	b	c	α	β	γ
1	-4681.30763	0.00000	0.00000	0.50491	6.1405	6.1183	5.7008	90.59	90.03	90.15
2	-4681.33761	0.00549	0.01102	0.50534	6.1345	6.1331	5.6846	90.37	89.97	90.14
3	-4681.34122	0.00536	0.01075	0.50535	6.1377	6.1353	5.6796	90.47	90.24	90.10
4	-4681.33171	0.01081	0.02167	0.50579	6.1306	6.1306	5.6962	90.23	90.23	89.91
5	-4681.32193	0.00592	0.01187	0.50579	6.1504	6.1032	5.7060	90.47	90.00	90.00
6	-4681.34105	0.00540	0.01084	0.50557	6.1546	6.1168	5.6851	90.28	89.92	90.23
7	-4681.35391	0.01139	0.02285	0.50560	6.1447	6.1217	5.6860	90.37	89.92	90.06
8	-4681.35247	0.01138	0.02282	0.50567	6.1454	6.1195	5.6874	90.37	90.16	90.04
9	-4681.32995	0.00426	0.00854	0.50586	6.1479	6.1280	5.6847	90.33	90.16	90.13
10	-4681.33085	0.00983	0.01972	0.50577	6.1375	6.1275	5.6952	90.22	90.19	89.91
11	-4681.34774	0.00964	0.01933	0.50572	6.1456	6.1396	5.6722	90.17	90.03	90.11
12	-4681.35079	0.01530	0.03069	0.50562	6.1369	6.1413	5.6790	90.16	90.32	90.00
13	-4681.33483	0.00473	0.00949	0.50587	6.1404	6.1335	5.6860	90.40	89.85	90.17
14	-4681.33731	0.01042	0.02090	0.50572	6.1369	6.1339	5.6898	90.38	90.08	89.82
15	-4681.35504	0.00978	0.01961	0.50554	6.1293	6.1521	5.6767	90.21	90.01	90.06
16	-4681.35552	0.01549	0.03107	0.50525	6.1224	6.1527	5.6836	90.15	89.94	89.90
17	-4681.34622	0.01549	0.03107	0.50542	6.1212	6.1562	5.6739	90.19	89.98	90.08
18	-4681.35152	0.00002	0.00004	0.50543	6.1246	6.1599	5.6674	90.27	90.28	90.01
19	-4681.35722	0.00501	0.01006	0.50423	6.1145	6.1729	5.6660	90.00	90.06	90.00
20	-4681.35006	0.00003	0.00007	0.50570	6.1420	6.1420	5.6726	90.10	89.90	90.10
21	-4681.36124	0.00587	0.01177	0.50541	6.1326	6.1448	5.6745	90.17	89.90	89.86
22	-4681.36205	0.00602	0.01206	0.50551	6.1337	6.1447	5.6735	90.19	90.20	89.83
23	-4681.36873	0.01095	0.02196	0.50447	6.1448	6.1215	5.6866	90.19	90.08	89.98
24	-4681.33183	0.00000	0.00000	0.50520	6.1329	6.1351	5.6806	90.09	90.28	90.11
25	-4681.36332	0.00000	0.00000	0.50535	6.1452	6.1452	5.6550	90.18	90.18	90.11
26	-4681.34634	0.00597	0.01196	0.50521	6.1360	6.1083	5.6977	90.26	90.10	90.01
27	-4681.37457	0.00584	0.01170	0.50438	6.1526	6.1313	5.6610	90.19	90.00	90.00
28	-4681.35977	0.00999	0.02003	0.50552	6.1493	6.1342	5.6704	89.99	90.01	89.81
29	-4681.34843	0.01584	0.03176	0.50572	6.1361	6.1280	5.6909	90.13	90.27	89.76
30	-4681.34366	0.00446	0.00893	0.50548	6.1446	6.1341	5.6757	89.79	90.18	89.80
31	-4681.36197	0.01005	0.02015	0.50632	6.1411	6.1404	5.6713	90.02	90.40	89.86
32	-4681.34666	0.01021	0.02047	0.50540	6.1327	6.1344	5.6872	90.21	89.80	90.18
33	-4681.36311	0.01586	0.03181	0.50563	6.1345	6.1404	5.6784	90.32	89.94	90.10
34	-4681.35962	0.00450	0.00903	0.50561	6.1395	6.1510	5.6660	89.98	89.95	90.10
35	-4681.34641	0.01014	0.02034	0.50545	6.1284	6.1452	5.6816	90.09	90.17	90.14
36	-4681.37895	0.00615	0.01232	0.50505	6.1336	6.1544	5.6575	90.00	90.18	90.00
37	-4681.36027	0.01189	0.02384	0.50486	6.1310	6.1285	5.6886	90.00	90.00	89.81
38	-4681.38958	0.01194	0.02394	0.50493	6.1413	6.1413	5.6627	90.01	89.99	89.78
39	-4681.35588	0.00000	0.00000	0.50531	6.1294	6.1304	5.6998	90.22	89.93	90.30
40	-4681.37332	0.00521	0.01046	0.50579	6.1391	6.1229	5.6960	90.00	90.00	90.00
41	-4681.37184	0.00566	0.01135	0.50506	6.1335	6.1363	5.6878	90.00	89.81	90.00
42	-4681.37572	0.01089	0.02184	0.50421	6.1373	6.1236	5.6964	90.00	90.00	90.13
43	-4681.31327	0.01764	0.03537	0.50516	6.1486	6.1486	5.6676	90.20	89.80	90.01
44	-4681.31578	0.02309	0.04630	0.50535	6.1498	6.1436	5.6708	90.20	89.99	89.99
45	-4681.33446	0.02861	0.05738	0.50538	6.1504	6.1504	5.6615	89.82	89.82	89.81

References

1 Nanda, K. D. & Beran, G. J. O. What governs the proton ordering in ice XV? *J. Phys. Chem. Lett.* **4**, 3165-3169 (2013).
